# Genome Wide Scan to Identify Potential Genomic Regions Associated With Milk Protein and Minerals in Vrindavani Cattle

**DOI:** 10.3389/fvets.2022.760364

**Published:** 2022-03-10

**Authors:** Akansha Singh, Amit Kumar, Cedric Gondro, A. K. Pandey, Triveni Dutt, B. P. Mishra

**Affiliations:** ^1^Animal Genetics Division, Indian Council of Agricultural Research-Indian Veterinary Research Institute, Bareilly, India; ^2^Department of Animal Science, Michigan State University, East Lansing, MI, United States; ^3^Livestock Production and Management, Indian Council of Agricultural Research-Indian Veterinary Research Institute, Bareilly, India; ^4^Division of Animal Biotechnology, Indian Council of Agricultural Research-Indian Veterinary Research Institute, Bareilly, India

**Keywords:** crossbred cattle, genome-wide association studies, milk minerals, milk protein, Vrindavani

## Abstract

In this study, genome-wide association study (GWAS) was conducted for identifying significantly associated genomic regions/SNPs with milk protein and minerals in the 96 taurine-indicine crossbred (*Vrindavani*) cows using 50K SNP Chip. After quality control, a total of 41,427 SNPs were retained and were further analyzed using a single-SNP additive linear model. Lactation stage, parity, test day milk yield and proportion of exotic inheritance were included as fixed effects in GWAS model. Across all traits, 13 genome-wide significant (*p* < 1.20 x 10^−06^) and 49 suggestive significant (*p* < 2.41 x 10^−05^) SNPs were identified which were located on 18 different autosomes. The strongest association for protein percentage, calcium (Ca), phosphorus (P), copper (Cu), zinc (Zn), and iron (Fe) were found on BTA 18, 7, 2, 3, 14, and 2, respectively. No significant SNP was detected for manganese (Mn). Several significant SNPs identified were within or close proximity to *CDH13, BHLHE40, EDIL3, HAPLN1, INHBB, USP24, ZFAT*, and *IKZF2* gene, respectively. Enrichment analysis of the identified candidate genes elucidated biological processes, cellular components, and molecular functions involved in metal ion binding, ion transportation, transmembrane protein, and signaling pathways. This study provided a groundwork to characterize the molecular mechanism for the phenotypic variation in milk protein percentage and minerals in crossbred cattle. Further work is required on a larger sample size with fine mapping of identified QTL to validate potential candidate regions.

## Introduction

Milk and dairy products are considered a nutrient-rich diet, consisting of fat, protein, lactose along with several essential micronutrients including vitamins and minerals. Minerals representing a small fraction (~9 g/L) of bovine milk, are important for nutritional and technological properties of milk (1). The minerals in milk are present either as an inorganic ion (soluble phase) or form colloidal complexes with organic matter such as proteins, carbohydrates, and ligands including amino acids and citrates (2, 3).

Minerals play an important role in several physiological and biological activities. For instance, calcium, phosphorus, and magnesium are involved in the development of connective tissues such as bones, muscles, cartilages, and teeth in humans (4). Calcium has been reported to play an important role in reducing cholesterol absorption, and in regulating blood pressure in humans (5). The zinc, manganese, iron, and copper are important components of several enzymes and play role in the immune system (6). These elements also serve as catalysts for many biochemical processes such as muscle contraction, nervous transmission, and nutrient absorption (7).

The concentration of milk minerals influences the stabilization of casein micelle in milk. Calcium, phosphorus, and magnesium are an important components of casein micelle and positively regulates the coagulation properties of the milk (8). The milk minerals are complex traits, influenced by both genetic and non-genetic factors, including nutrition, lactation stage, parity, season, and breed (9). Studies have shown that milk minerals have low to moderate heritability for Cu, Zn, Fe, and Mn (10); and moderate to high heritability for Ca, P, and Zn (1). This provides the possibility to alter the concentration of bovine milk minerals through selective breeding.

The genome-wide association studies may improve our understanding of the genetic architecture and underlying molecular mechanism for variation in protein and minerals content in bovine milk. Studies have revealed about several potential candidate genes associated with milk protein percentage in different cattle breeds (11, 12). However, for milk minerals only a limited number of genome-wide association studies has been reported (1, 3). Thus, the aim of this study was to identify genomic regions associated with milk protein percentage (%) and milk minerals including Ca, P, Cu, Zn, Fe, and Mn in Vrindavani cattle, a tropically adapted composite crossbred breed of dairy cattle of India.

## Materials and Methods

### Animals and Phenotypes

Morning milk samples (50 ml) were collected from 96 crossbred Vrindavani cattle of the ICAR-Indian Veterinary Research Institute, Bareilly. Vrindavani is a composite breed developed at Indian Veterinary Research Institute by crossing indicine (Hariana) with three taurine breeds (Holstein, Brown Swiss, and Jersey). Details of population structure and synthetic breed information of the Vrindavani population were described in a prior study by (13). The cows were kept in a loose housing system with free-stall dairy barn, fed *ad libitum* with mixed ration, and milked in a free-stall barn using automated milking systems. The cows were in milk (24-403 days) and from parity 1-8.

The protein percentage in milk was determined using a LactoScan milk analyzer (10). The milk minerals (Ca, P, Cu, Zn, Fe, and Mn) were estimated using Flame Atomic Absorption Spectrometer in technical triplicate samples. For the mineralization of milk samples, 5 ml of milk were dried in silica crucible using a hot-air oven. The dried samples were ashed using a muffle furnace. The ashed sample was mixed with 15 ml 1:1 diluted HCl and heated to dissolve the acid-soluble portion of total ash. The soluble portion was filtered and diluted to 100 ml using double distilled water. The digested samples were analyzed, using a flame atomic absorption spectrophotometer (14).

### Genotyping and Quality Control

Blood samples were collected from individual cows, with the approval from the Institutional Animal Ethics Commit- tee (IAEC) on ICAR-Indian Veterinary Research Institute, Bareilly. Genomic DNA was isolated using Qiagen DNeasy Blood Mini Kit (Qiagen, Valencia, CA) according to the manufacturer's instructions. The quality and quantity of DNA were evaluated using NanoDrop spectrophotometer, agarose gel electrophoresis and Qubit fluorometer. The extracted genomic DNA were genotyped using Illumina BovineSNP50 Bead Chip platform (Illumina Inc., San Diego, CA) consisting of 53,218 SNPs across the genome. The quality control of the SNP genotypes was performed using PLINK v 1.9 (15). SNPs with call rate <90%, minor allele frequency (MAF) <0.05 and significantly deviating from the Hardy-Weinberg equilibrium were excluded from the analysis. A total of 41,427 SNPs mapping autosomes were retained for further downstream analysis.

### Statistical Analyses

Prior to GWAS, the admixture profile and the level of exotic inheritance of individual animal was estimated using ADMIXTRE V.13.0 (16) using a reference panel of purebred taurine (Holstein-Friesian, Brown Swiss, and Jersey) and indicine (Hariana) cattle downloaded from WIDDE (http://widde.toulouse.inra.fr/widde/widde/main.do?module=cattle) and KRISHI (https://krishi.icar.gov.in/jspui/handle/123456789/31167) web portals. The fixed effect of lactation stage, parity, test day milk yield, and the proportion of exotic inheritance (percentage of Holstein-Friesian, Jersey and Brown Swiss, estimated by admixture analysis) were tested with *lm* function in R. A single-SNP linear regression of phenotype on genotype was fitted for GWAS analysis using snpStats package in R following Gondro (17). The genotypes were tested for additive effect using model.


$$\begin{array}{l} \text{y}=\text{X}\beta +\text{Z}\alpha +\text{e} \end{array}$$

Where *y* is the vector of phenotypic observations; *X* is an incidence matrix relating the phenotypes to the fixed effects including lactation stage, parity, test day milk yield, and the level of exotic inheritance computed by admixture analysis; β is the vector of fixed effects; *Z* is incidence matrix of genotypes (0 for the first homozygote AA; 1 for the heterozygote AB or BA; 2 for the second homozygote BB) of the fitted SNP; α is the vector of effects of the regression coefficient for SNPs, and *e* is the vector of residual effects with a normal distribution *N* ~ (0, *I*$\sigma_{e}^{2}$), where $\sigma_{e}^{2}$ is the residual variance.

The Bonferroni correction using 0.05/N, where “N” is the number of SNPs, was applied to the genome-wide significance threshold (18). The SNP effects were declared significant at a genome-wide level of *P* = 1.20 x 10^−06^ (0.05/41,427). Since Bonferroni correction was stringent, a suggestive significance threshold of *P* = 2.41 x 10^−05^ (1/41,427) was calculated (19). Association between individual SNPs and each trait are shown in the Manhattan plot using R.

The Ensembl database UMDv3.1 (https://www.ensembl.org/Bos_taurus/Info/Annotation) was used to search for genes flanking a region of 500 kb from the genome-wide significant and suggestive SNPs using BEDtools (20). Candidate genes close or containing significant SNPs were analyzed for functional enrichment using DAVID 6.8 (21). The significantly enriched pathways were identified based on enriched scores.

## Results and Discussion

The descriptive statistics for protein percentage and 6 individual minerals (Ca, P, Cu, Mn, Zn, and Fe) in the milk of Vrindavani cattle are presented in Table 1. A total of 9 (2 genome-wide and 7 suggestive) significant SNPs for protein percentage; and 53 (11 genome-wide and 42 suggestive) significant SNPs for six different mineral traits were identified.

**Table 1:** Descriptive statistics for protein percentage (%) and mineral (Ca, P, Cu, Mn, Zn, and Fe) in the Vrindavani milk.

**Traits**	**Mean**	**SD**	**Min**	**Max**	**CV**
Protein percentage(%)	2.814	0.193	2.480	4.170	6.859
Ca(mg/l)	1,471	589	681	2,960	40.015
P(mg/l)	1,192	319	602	2,360	26.762
Zn(mg/l)	3.694	1.526	1.130	7.773	41.310
Cu(mg/l)	0.892	0.709	0.130	2.213	79.442
Fe (mg/l)	8.925	4.423	3.180	22.400	49.560
Mn(mg/l)	0.794	0.350	0.180	1.930	44.121

### Protein Percentage

**Figure 1:**
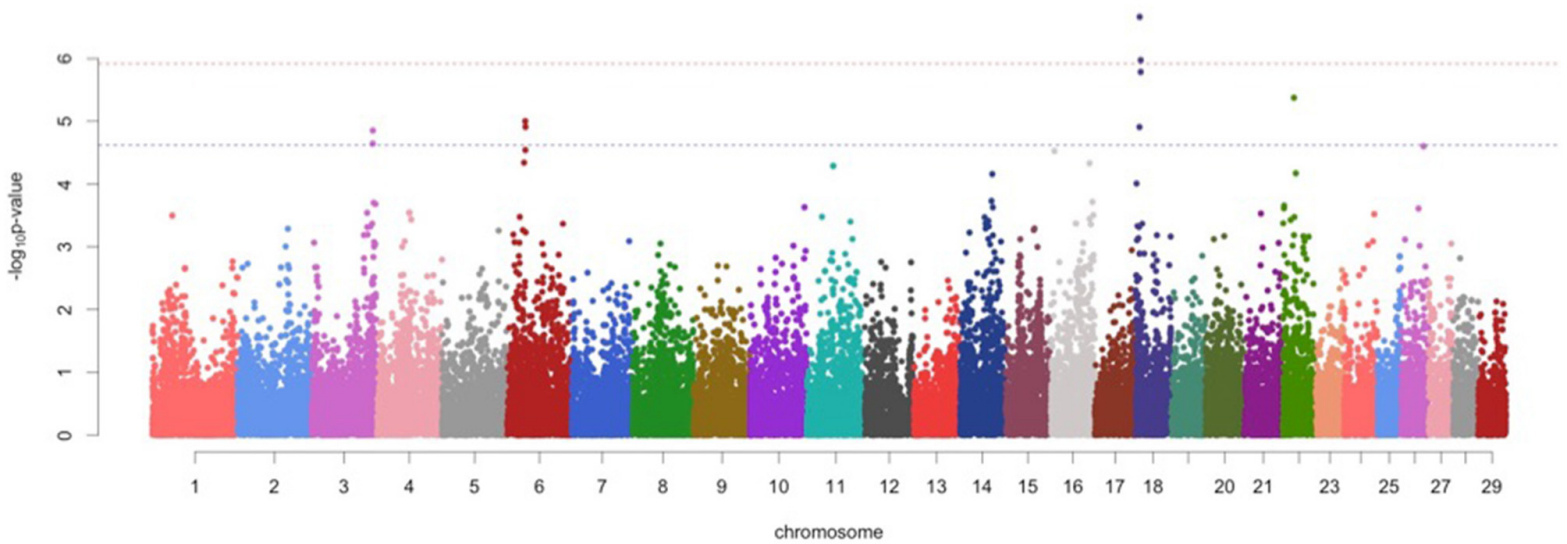
Manhattan plots for protein percentage in Vrindavani cattle milk. The red line represents genome-wide significant level (*p* < 1.20 x 10^−06^), and the blue line represents suggestive significant level (*p* < 2.41 x 10^−05^).

For protein percentage, two genome-wide significant SNPs (*p* < 1.20 x 10^−06^) and 7 suggestive significant SNPs (*p* < 2.41 x 10^−05^) were detected on BTA 3, BTA 6, BTA18, and BTA22 (Figure 1; Table 2). Of these, four significant SNPs found on BTA18 (7.63-99.15Mb) includes Beta-carotene oxygenase 1(*BCO1*), C-Maf inducing protein *(CMIP*), N-terminal EF-hand calcium binding protein 2 *(NECAB2*), Cadherin 13 (*CDH13*), oxidative stress induced growth inhibitor 1 (*OSGIN1*) and Solute carrier family 38 member 8 (*SLC38A8*) genes.

**Table 2:** Genome-wide and suggestive significant SNPs for protein percentage in Vrindavani milk.

**SNP**	**BTA**	**Position**	**P-value**	**Nearest candidate gene**
ARS-BFGL-NGS-73708	18	8037156	2.16 x 10^−07^	BCO1, CMIP
ARS-BFGL-NGS-85875	18	9948949	1.06 x 10^−06^	NECAB2, CDH13
ARS-BFGL-NGS-39549	18	9915057	1.62 x 10^−06^	OSGIN1, SLC38A8
Hapmap24079-BTA-136416	22	21000933	4.25 x 10^−06^	BHLHE40
Hapmap33287-BTC-032371	6	33026144	1.00 x 10^−05^	-
Hapmap33121-BTC-033043	6	33441527	1.24 x 10^−05^	-
Hapmap52589-rs29020496	18	7630875	1.24 x 10^−05^	CENPN, CMC2
ARS-BFGL-NGS-1337	3	113666410	1.41 x 10^−05^	USP 40, ATG16L1
BTB-00158861	3	113701688	2.28 x 10^−05^	MROH2A, UGT1A1

### Minerals

**Figure 2:**
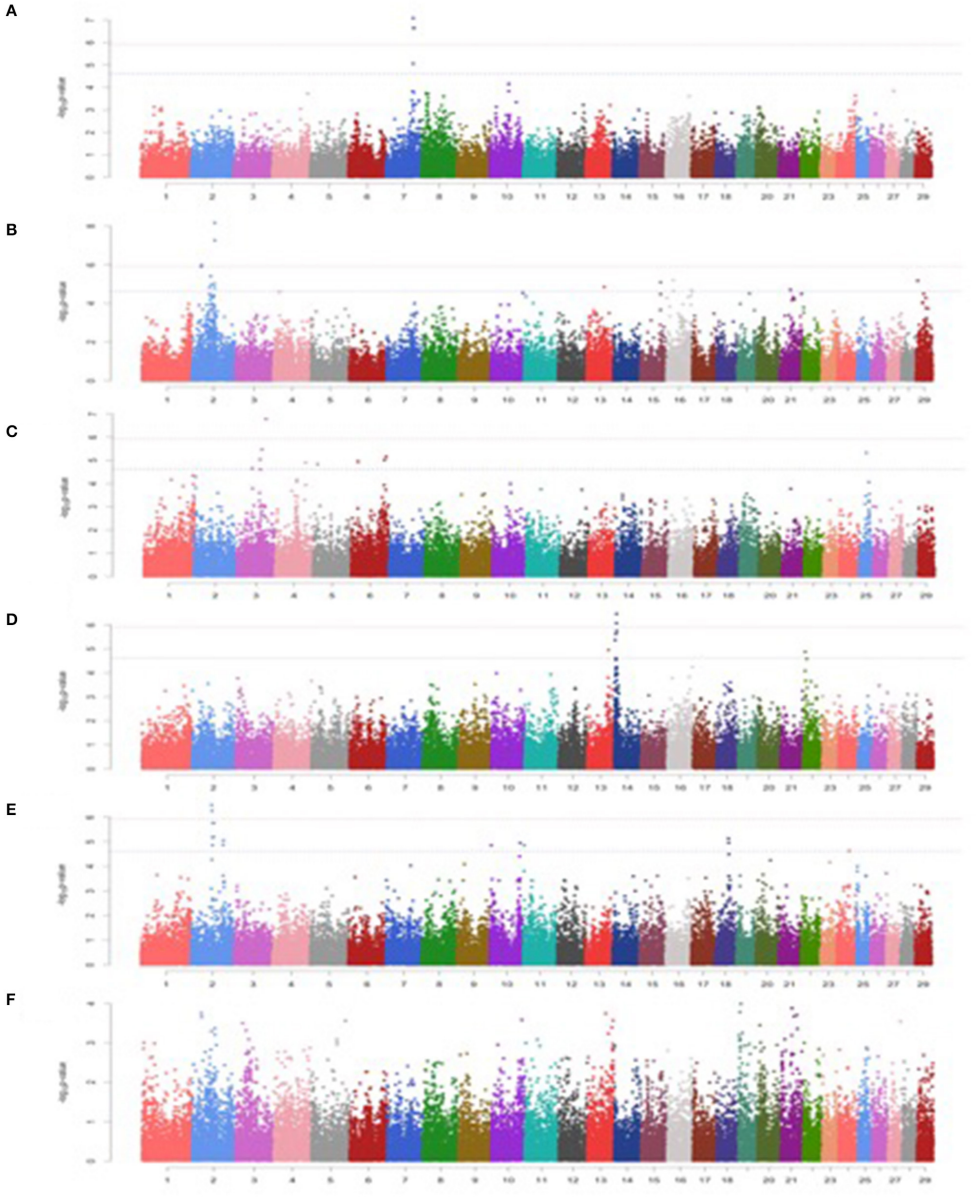
Manhattan plots for minerals in Vrindavani cattle milk. **(A)** Ca, **(B)** P, **(C)** Cu, **(D)** Zn, **(E)** Fe, **(F)** Mn. The red line represents genome-wide significant level (*p* < 1.20 x 10^−06^), and the blue line represents suggestive significant level (*p* < 2.41 x 10^−05^).

Among minerals, a total of 4, 17, 10, 7, and 15 significant SNPs were detected for Ca, P, Cu, Zn, and Fe, respectively. However, no significant SNP was detected for Mn (Figures 2A-F; Table 3).

**Table 3:** Genome-wide and suggestive significant SNPs for minerals (Ca, P, Cu, Zn, Fe, and Mn) in Vrindavani milk.

**Minerals**	**SNP**	**BTA**	**Position**	***P*-value**	**Nearest candidate gene**
Ca	BTB-01363189	7	86130978	8.20 x 10^−08^	HAPLN1, VCAN
Ca	BTB-00005259	7	86801082	2.29 x 10^−07^	EDIL3
Ca	BTB-01259879	7	89658972	2.29 x 10^−07^	RASA1
Ca	BTB-00326076	7	86044835	8.72 x 10^−06^	XRCC4
P	Hapmap45428-BTA-47987	2	72214554	6.89 x 10^−09^	INHBB,CFAP221
P	ARS-BFGL-NGS-85372	2	72369610	5.47 x 10^−08^	CFAP221
P	Hapmap39006-BTA-116188	2	30481073	1.03 x 10^−06^	SCN9A,SCN1A, SCN7A, TTC21B
P	BTB-01987495	2	29215002	1.27 x 10^−06^	XIRP2
P	BTB-01902301	2	59461527	3.91 x 10^−06^	HNMT
P	UA-IFASA-4860	16	20472340	6.17 x 10^−06^	ESRRG
P	ARS-BFGL-NGS-20615	29	5085168	6.67 x 10^−06^	NAALAD2
P	ARS-BFGL-NGS-105277	15	65530435	8.04 x 10^−06^	NAT10
P	ARS-BFGL-NGS-20993	2	70957217	9.92 x 10^−06^	EN1
P	ARS-BFGL-NGS-109852	2	61611083	1.08 x 10^−05^	CXCR4
P	ARS-BFGL-NGS-104967	13	55642499	1.44 x 10^−05^	CDH4
P	Hapmap38699-BTA-81626	2	55831708	1.55 x 10^−05^	-
P	ARS-BFGL-NGS-58619	2	71779928	1.60 x 10^−05^	CFAP221
P	ARS-BFGL-NGS-25171	21	34486610	2.01 x 10^−05^	ARID3B
P	Hapmap60572-rs29010980	16	78157760	2.15 x 10^−05^	CRB1
P	Hapmap49377-BTA-91839	16	21134492	2.28 x 10^−05^	ESRRG
P	ARS-BFGL-NGS-82451	16	81016831	2.37 x 10^−05^	CACNA1S
Cu	ARS-BFGL-NGS-1062	3	92082212	1.68 x 10^−07^	DHCR24, USP 24
Cu	BTB-01155381	3	79282515	3.40 x 10^−06^	INSL5
Cu	BTA-59703-no-rs	25	22197501	4.73 x 10^−06^	CACNG3
Cu	BTB-00280101	6	110508154	7.04 x 10^−06^	CLNK
Cu	BTA-107777-no-rs	3	73921609	9.19 x 10^−06^	-
Cu	ARS-BFGL-NGS-100916	6	105095758	9.61 x 10^−06^	PPP2R2C
Cu	BTB-01951920	6	21332376	1.12 x 10^−05^	PPA2
Cu	ARS-BFGL-NGS-116643	4	96258114	1.25 x 10^−05^	PLXNA4
Cu	Hapmap32870-BTA-162083	5	14189730	1.46 x 10^−05^	SLC6A15
Cu	ARS-BFGL-NGS-54036	3	48734443	2.24 x 10^−05^	ALG14,ABCD3
Zn	ARS-BFGL-NGS-76248	14	8879810	3.50 x 10^−07^	ST3GAL1
Zn	Hapmap31987-BTC-062044	14	8425401	8.59 x 10^−07^	ZFAT
Zn	UA-IFASA-7842	14	9472109	1.81 x 10^−06^	PHF20L1,KCNQ3
Zn	ARS-BFGL-BAC-10375	14	6616434	2.42 x 10^−06^	-
Zn	Hapmap26527-BTC-005059	14	4176618	4.31 x 10^−06^	AGO2, DENND3
Zn	ARS-BFGL-NGS-92308	13	67081069	1.13 x 10^−05^	MANBAL
Zn	ARS-BFGL-NGS-94105	22	7691228	1.34 x 10^−05^	FBXL2
Fe	ARS-BFGL-NGS-96204	2	65600025	3.26 x 10^−07^	NCKAP5
Fe	ARS-BFGL-NGS-32707	2	67078207	5.59 x 10^−07^	-
Fe	BTA-11592-rs29017351	2	69569936	1.75 x 10^−06^	CFAP221
Fe	ARS-BFGL-NGS-101411	2	72163562	1.75 x 10^−06^	INHBB
Fe	ARS-BFGL-NGS-22157	2	71906695	6.42 x 10^−06^	TMEM177
Fe	BTB-01536946	2	68429456	6.70 x 10^−06^	DPP10
Fe	ARS-BFGL-NGS-29914	18	42706451	7.38 x 10^−06^	DPY19L3
Fe	Hapmap40319-BTA-19899	2	102634206	8.86 x 10^−06^	VWC2L
Fe	ARS-BFGL-NGS-5074	18	43588701	1.10 x 10^−05^	RHPN2, FAAP24, LRP3
Fe	Hapmap43494-BTA-122491	10	94570405	1.12 x 10^−05^	-
Fe	ARS-BFGL-NGS-60714	2	102060979	1.33 x 10^−05^	IKZF2
Fe	Hapmap49519-BTA-19205	2	68058489	1.39 x 10^−05^	DPP10
Fe	ARS-BFGL-NGS-113875	11	3106988	1.41 x 10^−05^	ACTR1B, ZAP70
Fe	ARS-BFGL-NGS-40198	10	2617295	1.41 x 10^−05^	YTHDC2
Fe	Hapmap57118-rs29009938	24	39365195	2.35 x 10^−05^	EPB41L3

A total three genome-wide significant and 1 suggestive significant SNPs on BTA7 (86.04-89.65Mb) were found to be associated with Ca. The region contains several genes including Hyaluronan and proteoglycan link protein 1 (*HAPLN1*), Versican (*VCAN*), EGF like repeats, and discoidin domains 3 (*EDIL3*). A total of three genome-wide and 14 suggestive significant SNPs were found to be associated with P. The most significantly (*p* = 6.89 x 10^−09^) associated SNP (Hapmap45428-BTA-47987) was located 0.10 Mb downstream from Inhibin subunit beta B (*INHBB*) gene and 0.81 Mb downstream from Cilia and flagella associated protein 221 (*CFAP221*) gene on BTA2. Total 1 genome-wide and 9 suggestive significant SNPs were observed to be associated with Cu. The most significantly (*p* = 1.68 x 10^−07^) associated SNP (ARS-BFGL-NGS-1062) with Cu was identified at 0.63 Mb downstream from the 24-dehydrocholesterol reductase (*DHCR 24*) gene on BTA3. The SNP (BTA-59703-no-rs) significantly associated with Cu (*p* = 4.73 x10^−06^) on BTA25 was located within Calcium voltage-gated channel auxiliary subunit gamma 3 (*CACNG3*) gene. A total of 2 genome-wide and 5 suggestive significant SNP were associated with Zn. Out of these 7 SNPs, 5 significant SNPs were located within 5.29 Mb (4.17-9.47 Mb) region of BTA14. The most significant (*p* = 3.50 x 10^−07^) SNP (ARS-BFGL-NGS-76248) associated with Zn was located 0.09 Mb upstream from the ST3 beta-galactoside alpha-1 (*ST3GAL1*) gene. A total of 2 genome-wide and 15 suggestive significant SNPs were significantly associated with Fe. Total six significant SNPs were identified within 4.10 Mb region (65.60-72.17 Mb) on BTA2. The most significant (*p* = 3.26 x 10^−07^) SNP (ARS- BFGL-NGS-96204) associated with Fe was located 0.45 Mb away from *NCKAP5* gene on BTA2. The most significantly (*p* = 1.02 x 10^−04^) associated SNP (Hapmap58269-rs29018185) with Mn was located on BTA 19, although it was below the cut-off threshold level of significance.

The functional enrichment of genes close to significant SNPs was involved in ion transport (GO:0005248), metal ion binding (GO:0005509), integral and transmembrane protein (GO:0086010), and signaling pathway (Supplementary Table S1).

In the present study, several significant SNPs found to be associated with protein percentage and individual mineral concentration in the milk of Vrindavani cattle. To our knowledge, this is the first study to identify genomic regions associated with milk mineral content in composite Vrindavani cattle. Detection of several previously reported genes and genomic regions associated with different milk composition traits indicates their potential role in regulating the concentration of minerals and protein percentage in bovine milk.

For protein percentage, the significant 1.9 Mb region (8.03-9.94 Mb) on BTA 18 was partly overlapping with previously reported QTLs associated with milk coagulation (22). The milk coagulation is directly influenced by the casein composition, which indicates that this region may have the potential to influence the protein percentage in milk (23). This region includes the *CDH 13* (Cadherin-13) gene which is expressed in mammary tissues and associated with the protein content of the milk (24).

On BTA2, 18 significant SNPs were identified for P and Fe content. Two significant SNPs associated with P and Fe were 0.10 and 0.35 Mb away from the inhibin subunit beta B (*INHBB*) gene on BTA2. In humans, *INHBB* gene plays a major role in regulating calcium and phosphorus during bone formation (25). More specifically, *INHBB* facilitates interaction with the beta glycan, which stimulates extracellular matrix mineralisation (26). The SNP associated with Fe is located 0.35 Mb downstream from the IKAROS family zinc finger 2 (*IKZF2*) is involved in metal ion binding (27) and host immune response (28).

On BTA3, three significant SNPs were associated with milk Cu content. Buitenhuis et al. (1) identified several significant SNPs for Cu on BTA3 (91.0-91.3 Mb) in Danish Holstein milk. However, these SNPs do not overlap with the significant SNPs for Cu identified in our study. The most significant SNP associated with Cu is located 0.79 Mb upstream from the Ubiquitin Specific Peptidase 24 (*USP24*) gene. The *USP24* gene belongs to the cysteine proteases family and plays role in protein deubiquitination of the proteins which influences the stability of the casein micelle in the milk (29). However, the role of this gene in the regulation of milk Cu concentration is not known.

On BTA7, four significant SNPs associated with Ca, were located close to *HAPLNI, VCAN*, and *EDIL3* genes. These genes are referred to as calcium-binding proteins (GO:0050850, positive regulation of calcium-mediated signaling) and are involved in tissue calcification and bone mineralization in vertebrates (30). In chickens, the EGF-like repeats and discoidin domains 3 (*EDIL3*) gene bind with the calcium ion to guide vesicular transportation of minerals during eggshell calcification (31). Even though its role in milk calcium is not known, *EDIL3* could be considered as a candidate gene, which warrants fine mapping.

The SNP named Hapmap3 1987-BTC-062044 was significantly (*p* = 3.50 x 10^−07^) associated with Zn, is located at 0.12 Mb away from the Zinc finger and AT-hook domain containing (ZFAT) on BTA14, which is involved in the transcription of immune-related genes.

## Conclusion

Our study identified several candidate genes associated with milk protein percentage and milk minerals, that are involved in ion transport, signaling pathways *via* integral protein, transmembrane membranes, zinc-fingers, and metal ion binding. The strongest association for protein percentage was identified on BTA18. Among studied minerals, the strongest association for Ca, P, Cu, Zn, and Fe were found on BTA 7, 2, 3, 14, and 2, respectively. These roles of genes and genomic regions suggested that milk mineral concentration is probably regulated by transportation and homeostasis of ions. These identified variants are a step forward to characterize the molecular mechanism affecting milk minerals in Vrindavani cattle. However, additional validation of detected variants and their association with milk minerals is required on large sample size.

## Data Availability Statement

The datasets presented in this study can be found in online repositories. The names of the repository/repositories and accession number(s) can be found at: Figshare, https://figshare.com/articles/dataset/Genotypes/12808343/2.

## Ethics Statement

The animal study was reviewed and approved by Institute Animal Ethics Committee of Indian Veterinary Research Institute, Bareilly.

## Author Contributions

AK conceived and designed the experiments. AS and AK performed the experiments, analyzed the data, and wrote the paper. CG, AP, and TD contributed reagents/materials/analysis tools. All authors contributed to the article and approved the submitted version.

## Funding

This project work was supported by the CAAST-ACLH project of NAHEP. AS was recipient of fellowship from ICMR fellowship during her PhD programme.

## Conflict of Interest

The authors declare that the research was conducted in the absence of any commercial or financial relationships that could be construed as a potential conflict of interest.

## Publisher's Note

All claims expressed in this article are solely those of the authors and do not necessarily represent those of their affiliated organizations, or those of the publisher, the editors and the reviewers. Any product that may be evaluated in this article, or claim that may be made by its manufacturer, is not guaranteed or endorsed by the publisher.
